# Temporal Gene Expression Profiling during Rat Femoral Marrow Ablation-Induced Intramembranous Bone Regeneration

**DOI:** 10.1371/journal.pone.0012987

**Published:** 2010-10-01

**Authors:** Joel K. Wise, Kotaro Sena, Karen Vranizan, Jacob F. Pollock, Kevin E. Healy, W. Frank Hughes, D. Rick Sumner, Amarjit S. Virdi

**Affiliations:** 1 Department of Anatomy and Cell Biology, Rush University Medical Center, Chicago, Illinois, United States of America; 2 Department of Molecular and Cell Biology, Helen Wills Neuroscience Institute and Functional Genomics Laboratory, University of California, Berkeley, California, United States of America; 3 Department of Bioengineering, University of California, Berkeley, California, United States of America; 4 Department of Materials Science and Engineering, University of California, Berkeley, California, United States of America; New Mexico State University, United States of America

## Abstract

Enhanced understanding of differential gene expression and biological pathways associated with distinct phases of intramembranous bone regeneration following femoral marrow ablation surgery will improve future advancements regarding osseointegration of joint replacement implants, biomaterials design, and bone tissue engineering. A rat femoral marrow ablation model was performed and genome-wide microarray data were obtained from samples at 1, 3, 5, 7, 10, 14, 28, and 56 days post-ablation, with intact bones serving as controls at Day 0. Bayesian model-based clustering produced eight distinct groups amongst 9,062 significant gene probe sets based on similar temporal expression profiles, which were further categorized into three major temporal classes of increased, variable, and decreased expression. Osteoblastic- and osteoclastic-associated genes were found to be significantly expressed within the increased expression groups. Chondrogenesis was not detected histologically. Adipogenic marker genes were found within variable/decreased expression groups, emphasizing that adipogenesis was inhibited during osteogenesis. Differential biological processes and pathways associated with each major temporal group were identified, and significantly expressed genes involved were visually represented by heat maps. It was determined that the increased expression group exclusively contains genes involved in pathways for matrix metalloproteinases (MMPs), Wnt signaling, TGF-β signaling, and inflammatory pathways. Only the variable expression group contains genes associated with glycolysis and gluconeogenesis, the notch signaling pathway, natural killer cell mediated cytotoxicity, and the B cell receptor signaling pathway. The decreased group exclusively consists of genes involved in heme biosynthesis, the p53 signaling pathway, and the hematopoietic cell lineage. Significant biological pathways and transcription factors expressed at each time point post-ablation were also identified. These data present the first temporal gene expression profiling analysis of the rat genome during intramembranous bone regeneration induced by femoral marrow ablation.

## Introduction

Bone is a dynamic organ that undergoes continuous remodeling by controlled cycles of bone resorption and bone formation, which are balanced to preserve bone mass. In the case of common metabolic bone disorders, such as osteoporosis, reduction in skeletal mass is caused by an imbalance between bone resorption and bone formation. Both types of bone regeneration, intramembranous and endochondral in response to fracture healing, are generally known to have parallels with developmental bone formation, and involve distinct yet interdependent healing phases consisting of biologically complex processes regulated by a very large number of transcriptional events[1]–[3]. Bone marrow ablation in long bones induces intramembranous bone formation and subsequent bone resorption in order to regenerate normal bone marrow, and was originally established as an experimental model to study hematopoiesis[4], [5]. Several groups, including ours, have used the rat marrow ablation model for investigations related to implant fixation[6]–[12]. The marrow ablation model has been utilized further as an experimental model to study intramembranous bone regeneration with concentration on histological and biochemical approaches and focused gene expression analysis[2], [13]–[16]. From these investigations, it is understood that following marrow ablation there are three major and distinct, yet overlapping, phases of healing which can be generally described. The first phase primarily consists of clot formation and inflammation from days 1 to 5. The second major phase is that of repair from day 3 to 14, and involves neovascularization, perivascular maturation, mesenchymal stem cell migration, proliferation and condensation, osteoblastic differentiation, and woven bone formation. Lastly, there is a remodeling phase from approximately days 10 to 28, until restoration of fatty and hematopoietic marrow is achieved by 56 days.

Although the distinct phases of inflammation, bone repair, and bone remodeling following rat femoral ablation surgery can be well-defined by histological methods, enhanced understanding of temporal gene expression profiling and identification of significant biological pathways associated with the distinct phases of intramembranous bone regeneration will greatly improve future advancements of fixation and osteointegration of joint replacement implants[17], design and synthesis of novel biomaterials, and bone tissue engineering[18].

Therefore, the mechanical ablation of the rat femoral marrow cavity is an established and suitable model for gene expression profiling studies regarding intramembranous bone regeneration. Our group has previously used this rat femoral marrow ablation model to characterize coexpression patterns of 39 genes during repair phases of intramembranous bone regeneration up to 14 days[2] and to report modulation of expression of 21 osteogenic genes following local application of rhTGF-β2[19]. To date, there have been no published reports of genome-wide temporal transcriptional analysis of intramembranous bone regeneration that takes place in a marrow ablation model. A recent report utilized microarray data and temporal transcriptional profiling analysis of the mouse transcriptome of an endochondral bone formation process occurring during a 21 day period of fracture healing[20]. Certain gene expression data analysis methods used for that study are similarly used in the present study, including a Bayesian modeling approach to cluster temporal gene expression profiles providing by the Cluster Analysis of Gene Expression Dynamics Program (CAGED), and specific analysis to identify significant biological processes and pathways using the DAVID bioinformatics resources. In this study, we identify significant pathways including matrix metalloproteinases (MMPs), Wnt signaling, TGF-β signaling, and notch signaling for major temporal expression groups, as well as differential pathways and transcription factors expressed at each time point up to 56 days.

## Materials and Methods

### Rat Model

In an Institutional Animal Care and Use Committee of the Rush University Medical Center (IACUC; Protocol #06-005) approved study, 45 adult male rats (Sprague Dawley, 400-425 g) were divided into nine groups: intact control (0 day), and 1, 3, 5, 7, 10, 14, 28, and 56 days post marrow ablation. Forty animals received unilateral femoral ablation, adapting the method described previously[2], [14], [15]. Briefly, the rats were anesthetized by intraperitoneal injection of ketamine hydrochloride (100 mg/kg) and xylazine (5 mg/kg) supplemented as necessary. Surgery was performed with adherence to aseptic technique. The hindlimb was shaved and scrubbed with ethanol and betadine solution. An incision (∼1 cm) was made along the medial aspect of the patella, and the patella along with the quadriceps tendon and patellar ligament were retracted to expose the distal condyles of the femur. A 2.0 mm hole was drilled through the patellar surface of the femur to gain access to the medullary canal. The contents of the marrow cavity were disrupted mechanically and reamed by hand with a 2.0 mm drill bit proximally up to the lesser trochanter. The canal was then irrigated with 10 ml of saline. The distal opening hole in the bone was sealed with bone wax, the patella was repositioned, and the deep fascia and skin sutured separately. Following surgery, the locomotion, grooming, and eating habits of the surgically treated rats were normal. At each time point, animals were killed in a carbon dioxide chamber, and whole femurs were harvested and denuded of soft tissue.

### Total RNA Extraction

For three samples per time point, both the distal and proximal ends of the femurs were cut off in order to exclude the epiphyses and growth plate regions. Diaphyseal marrow tissue and cells from the mid-shaft were flushed out using Trizol®. Following homogenization, 1 ml of solution was transferred to a 1.5 ml Eppendorf tube and centrifuged at 12,000 g for 10 minutes at 4°C to remove insoluble material. The supernatant containing RNA was collected, mixed with 0.2 ml of chloroform, and centrifuged at 12,000 g for 15 minutes at 4°C. After RNA in the aqueous phase was transferred into a new tube, the RNA was precipitated by mixing 0.5 ml of isopropyl alcohol and recovered by centrifuging the tube at 12,000 g for 10 minutes at 4°C. The RNA pellet was washed briefly in 1 ml of 75% ethanol and centrifuged at 7,500 g for 5 minutes at 4°C. Finally, the total RNA pellet was dissolved in diethylpyrocarbonate (DEPC) water, and its quantity was assessed by spectrophotometric analysis.

### Experimental Design for Gene Expression Profiling

**Figure 1:**
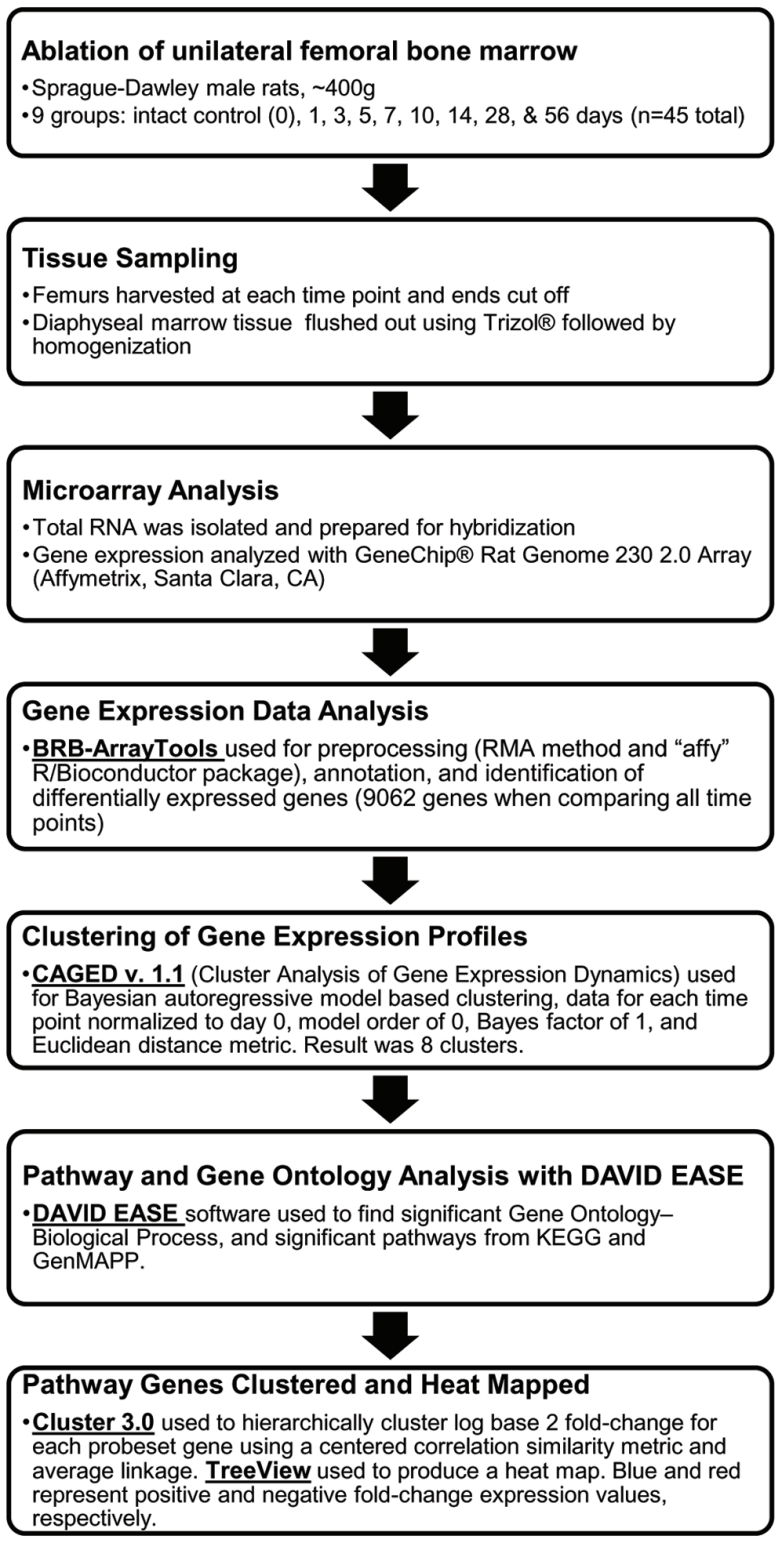
Flow chart of the chronological steps involved in the microarray analysis. Each box and corresponding arrow display a major step in the experimental design and genome-wide analysis using microarrays and gene expression analysis resources. Programs used for certain steps of the analysis are bold and underlined.

A flow chart displaying the major steps involved in the experimental design is provided in **Figure 1**. Gene expression profiling was performed on total RNA samples that were collected at day 0, which is the biological reference of intact bone, and at 1, 3, 5, 7, 10, 14, 28, and 56 days post-ablation of rat femoral marrow.

### Microarray Hybridization and Data Acquisition

For each of the three samples per time point, gene expression was analyzed with GeneChip® Rat Genome 230 2.0 Arrays (Affymetrix, Santa Clara, CA), which is comprised of over 31,000 probe sets representing approximately 28,700 well-characterized rat genes. For each gene, eleven pairs of oligonucleotide probes are synthesized *in situ* on the arrays. Total RNA from the samples was hybridized using optimal reagents and standardized protocols, a GeneChip® Hybridization Oven, a GeneChip® Fluidics Station, and a GeneChip® Scanner 3000 enabled for High-Resolution Scanning. Data acquisition from the microarrays also required the use of Affymetrix GeneChip® Command Console® Software (AGCC).

### Microarray Expression Data Pre-processing

The raw gene expression data from Affymetrix were available in the form of binary CEL (Affymetrix cell intensity) files. The open-source integrated software system BRB-ArrayTools (Dr. Richard Simon and BRB-ArrayTools Development Team) [21] was used and the CEL files were collated with RMA (robust multi-array average) [22] method and “affy” R/Bioconductor package to compute probeset summaries [23]. This utilized a three-step approach of background correction on PM (Perfect Match) data, quantile normalization, and Tukey's median polish algorithm for summarization of probe level data. The data discussed in this publication have been deposited in NCBI's Gene Expression Omnibus[24] and are accessible through GEO Series accession number GSE22321 (http://www.ncbi.nlm.nih.gov/geo/query/acc.cgi?token=hbybhuqmksgeydy&acc=GSE22321). Specific genes are mentioned by the gene name and/or official gene symbol, along with the Entrez Gene ID number in square brackets.

### Microarray Expression Data Analysis

A univariate F-test (with random variance model) with a significance threshold of p<1×10^−3^ (an appropriately stringent significant level to reduce the chance of false positives) was performed with BRB-ArrayTools and used to determine differentially expressed gene probe sets over all time points. Gene expression profiles were clustered with Cluster Analysis of Gene Expression Dynamics (CAGED version 1.1) program, which utilizes a Bayesian model-based clustering method on temporal gene expression data[25] and uses an agglomerative procedure to identify the most probable set of clusters, where genes assigned to certain clusters have similar temporal expression profiles. In turn, genes clustered together in this way are likely to share similar physiological functions or regulation. Data were normalized as ratios to the expression values on Day 0. A model order of 0 was used, where data from each time point are assumed to be independent from each other. The prior precision and gamma value were set to 1 and 0, respectively, where the prior precision is the size of the sample upon which the prior distribution is built, while the gamma value is the rate to zero of the prior precision, with 0 representing the case of perfect ignorance. A Bayes Factor of 1 was used to impose this minimum limit for accepting the merging of two clusters if the Bayes Factor of their merging is at least the value of 1. The method required a similarity measure to guide the search procedure and a Euclidean distance measure between gene expression profiles was adopted. Goodness of fit of the resulting model was assessed by checking the normality of the standardized residuals of each cluster.

Each cluster identified by CAGED analysis was assigned to one of the three major temporal groups according to increased, variable, or decreased expression, which were then further analyzed with DAVID EASE (version 2.0)[26] software to identify significant gene ontology categories or biological pathways. All significant gene categories associated with the Biological Processes domain of the Gene Ontology (GO) Consortium[27] were determined, using a significance threshold of p<0.05. Overall major categories of biological processes were formed by manually combining specific subcategory terms having related or overlapping functions. DAVID EASE was also used to analyze the gene sets for each major temporal group (increased, variable, decreased expression) to find significant known pathways determined by KEGG[28] and GenMAPP[29] databases, using a significance threshold of p<0.05. Cluster 3.0[30] was used to cluster the log base 2 expression values (for each day time point vs. day 0 time point) of genes associated with certain significant biological pathways of interest from the three major temporal expression groups (increased, variable, and decreased expression). For each significant pathway identified, the number of significant genes from the initial significant gene list known to be associated with a given pathway is noted as “Gene List Hits”. Additionally, the total number of genes on the Affymetrix GeneChip® Rat Genome 230 2.0 Array known to be associated with a given pathway is noted as “Gene Total Hits”. The log base 2 fold-change ratios were clustered using hierarchical clustering with a centered correlation distance/similarity metric and average linkage clustering method. The clustered data table file was viewed in TreeView[31] using the pixel setting contrast default of 3 and using blue and red to represent positive and negative fold-change expression values, respectively. A univariate two-sample T-test with significance threshold of p<1×10^−3^ in BRB-ArrayTools was further used to determine significant gene lists of gene probe sets differentially expression on each time point (day 1, 3, 7, 10, 14, 28, and 56 vs. day 0 intact control) post-ablation. DAVID EASE was used to analyze gene lists for each time point post-ablation and find all known pathways determined by KEGG and GenMAPP databases using a significance threshold of p<0.05. Additionally the total number of probe sets known to be transcription factors and present on the GeneChip® Rat Genome 230 2.0 Array was determined to be 1,254, and significant gene lists for each time point (each day vs. day 0) were compared with the total number of transcription factor list, and the number of significant transcription factors expressed for each time point was found.

### Histological methods for imaging of femoral marrow samples at each post-ablation time point

For two animals per time point, the whole femur was dissected and fixed in 4% paraformaldehyde in phosphate-buffered saline. Tissues were decalcified with 0.5 M ethylenediaminetetraacetate (EDTA) and embedded in paraffin. Four-µm-thick sections were cut in the sagittal plane through the femur and stained with hematoxylin and eosin. Images from selected sub-metaphyseal sites were recorded at 10x and 40x magnification with a NikonH600L photomicroscope.

## Results

### Representative histological images of femoral marrow samples at each time point post-ablation

**Figure 2:**
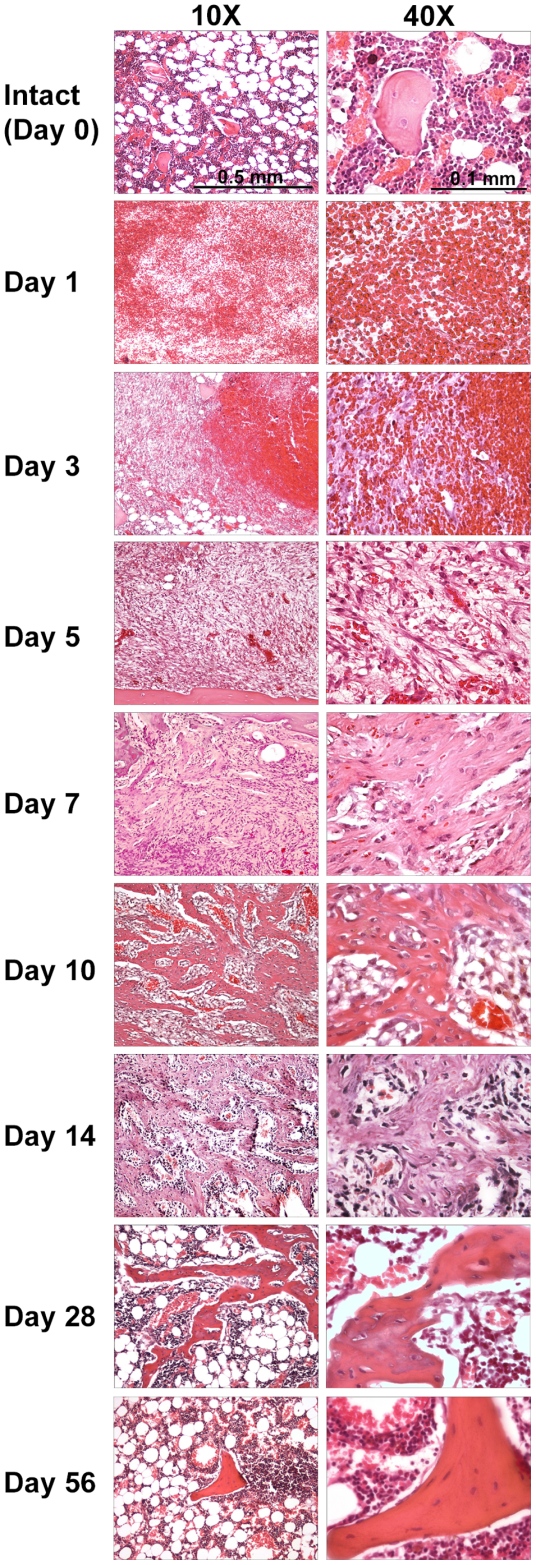
*In-situ* histology of intramedullary bone formation at each post-ablation time point (10X & 40X). **Intact Day 0,** submetaphyseal regions of the intact bone characteristically are populated by adipocytes and hematopoetic cell populations among scattered trabecular profiles. **Day 1**, the post-ablation clot containing scattered polymophonuclear cells fills the marrow space. **Day 3**, the clot is beginning to show cellular infiltration by cells of indeterminate origin. **Day 5**, organization of the clot with cell transformations in a fibrovascular structure with small vessels and an immature collagenous network. **Day 7** and **Day 10**, increases in collagenous interstitial matrix. **Day 14**, consolidation and modeling of pretrabecular matrix structure with expansion of cellularity in perivascular space of the maturing vascular network. **Day 28** and **Day 56**, maturation of trabecular structure and marrow shows reconstitution of the pre-ablation tissue architecture. Osteocytes and bone-lining osteoblastic cells are evident.

Stages of bone regeneration in the ablated intramedullary space are illustrated in **Figure 2**. This regenerative sequence occurs in spatially and temporally complex domains within the metaphyseal and diaphyseal regions. The process can be divided into eight subphases. Immediately following the ablation, a blood clot fills the space producing a fibrin and platelet laden substrate for a succession of cellular infiltrates (clot consolidation phase, days 1–3). The perimeter of this clot shows polymorphonuclear inflammatory infiltration associated with damaged penetrating vessels (inflammatory phase, days 3–5). Subsequently, there is a rapid invasion of fibrovascular progenitors that actively generate a collagenous matrix within the clot (granulation phase, days 4–7). Vasculogenesis and angiogenesis become evident in this primitive matrix structure wherein new capillary networks become connected to feeder vessels to establish a circulation (neovascularization phase, days 3–7). Perivascular infiltrates, including pericytes, lymphocytes, mast, and mononuclear cells become prominent as this primitive vascular network matures and establishes flow (perivascular maturation phase, days 5–10). Giant cells become conspicuous within this perivascular domain. The extravascular collagenous domains increase in density with sites of mineralization characteristic of woven-bone that assumes trabecular form (osteogenic phase, days 5–10). Arrays of osteoblastic cells begin to occupy the woven bone surfaces to produce lamellar bone on the woven bone cores (trabecular maturation phase, days 7–14). Variable sites of osteoclastic activity accompany this trabecular maturation phase, finally tipping the balance to the remodeling and resorption of the intramedullary trabecular structure (resorption phase, days 10–28) as the fatty and hematopoietic marrow is reconstituted (marrow reconstitution phase, days 28–56). The stages represented in the figure are overlapping as new trabecular bone rapidly forms to occupy the intramedullary space by day 7 to 14 and, following resorption of much of this bone by day 28, the fatty and hematopoietic marrow composition is restored.

### Temporal clusters of expression profiles

**Figure 3:**
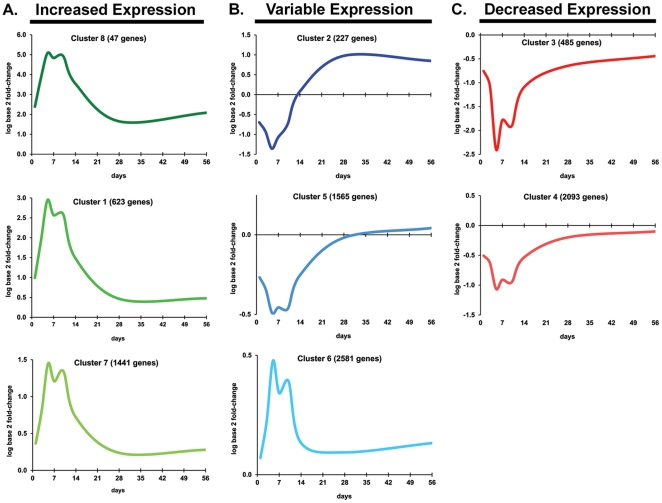
Expression profiles of temporal clusters during intramembranous bone regeneration. Clustering of expression profiles of the 9,062 statistically significant gene probe sets by Bayesian modeling produced 8 temporal clusters, which were further grouped into three major temporal groups of **(A) Increased Expression** (cluster #1, 7, and 8; graphed as green), **(B) Variable Expression** (cluster #2, 5, 6; graphed as blue), and **(C) Decreased Expression** (cluster #3 and 4; graphed as red). Data is presented as log base 2 fold-change values over the time points of day 1, 3, 5, 7, 10, 14, 28, and 56 days post-ablation, and is graphed as a solid line. The cluster number and the number of genes for each clustered gene expression profile graph are denoted in the title. Note that the scale for the vertical axis is not constant.

The graphical representations of the average expression profiles for each of the 8 unique temporal clusters determined by cluster analysis of significant gene probe sets are shown in **Figure 3**. These clusters can be differentiated according to the pattern and degree of differential expression. Clusters 1, 7, and 8 were assigned to the increased expression group (**Figure 3A**), clusters 2, 5, and 6 were assigned to the variable expression group (**Figure 3B**), and clusters 3 and 4 were assigned to the decreased expression group (**Figure 3C**). Clusters were assigned to the variable group if the expression profiles of all genes contained in that cluster exhibited patterns of both increased and decreased expression temporally (namely cluster 2 and 5) or if the log base 2 fold-change of expression values were less than 0.5 for all time points (cluster 6). **Table S1** provides detailed information and expression data regarding the gene probe sets comprising each of the 8 clusters.

### Identification of significant biological process ontologies and pathways associated with major temporal groups of clustered gene expression profiles

**Figure 4:**
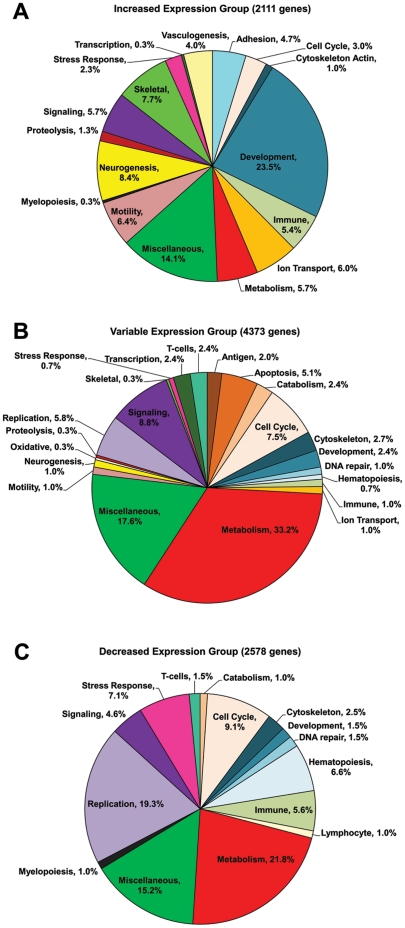
Percentage distribution of significant Gene Ontology/Biological Processes associated with each major group of clustered gene expression profiles. DAVID EASE (version 2.0) software was used to analyze the gene lists for each major temporal group (increased, variable, and decreased expression) to find all categories associated with the Biological Processes domain of the Gene Ontology (GO) consortium, using a significance threshold of p<0.05. Individual gene ontology results with related or overlapping functions were manually combined into overall major categories. Each biological process category is assigned a distinct color, and consistent colors were used for comparisons of the same biological process category found for more than one of the three major groups of clustered gene expression profiles/each of the three pie graphs, which include Increased Expression Group shown in **Figure 4A**, Variable Expression Group shown in **Figure 4B**, and Decreased Expression Group shown in **Figure 4C**. For example, the major category of “metabolism” is consistently assigned the color red. The number of genes within each of the three major temporal groups (increased, variable, and decreased expression) that underwent gene ontology/biological process analysis is denoted in the title of each pie graph.

Pie graphs (**Figure 4**) present the percentage distribution of statistically significant (p<0.05) biological process ontologies identified for increased, decreased, and variable expression groups. All individual biological ontology results were manually examined for related or overlapping functions in order for consolidation into overall major categories of biological process and this detailed information is summarized and shown for each major temporal group in **Table S2A-D**. For all three of the major temporal groups and therefore during all time points of intramembranous bone regeneration following marrow ablation, certain biological processes comprise large percentages out of the total biological functions identified. These aforementioned biological processes were specific aspects of metabolism, a large variety of developmental related processes, cell cycle, cellular signaling, as well as a large category referred to as miscellaneous. In contrast, there are unique differences in the percentage distributions of specific biological functions identified in each of the three temporal expression groups, and these will be described henceforth.

The major biological processes identified exclusively for increased gene expressions, which all peaked between days 5 and 10 as shown by their average expression profiles (**Figure 4A**), consisted of a large variety of processes related to neurogenesis, skeletogenesis, cell motility, cell adhesion, vasculogenesis, and Wnt signaling, as supported by detailed information in **Tables S2A** and **S2B**. Biological processes identified for the variable expression group (**Figure 4B**) were dominated mostly by elements of metabolism but also processes related to DNA replication, apoptosis, cytoskeleton, catabolism, DNA transcription, and T-cell functions.

Likewise, the majority of the biological processes found for the decreased expression group (**Figure 4C**) were related to metabolism and DNA replication, however, other notable percentage distributions were exclusively identified for hematopoiesis and myelopoiesis.

Another more focused method of analysis was performed on the gene expression data of the three major temporal groups (increased, variable, and decreased expression). Significant (p<0.05) known biological pathways determined by KEGG and GenMAPP databases were identified and are presented in **Table 1**. This analysis revealed that certain significant pathways identified were unique to the gene expressions comprising only one of the three major temporal groups. Specifically, the increased expression group exclusively contains genes involved in pathways for matrix metalloproteinases (MMPs), Wnt signaling, axon guidance, TGF-β signaling, and inflammation. Contrastingly, only the variable expression group contains genes associated with glycolysis and gluconeogenesis, notch Signaling Pathway, natural killer cell mediated cytotoxicity, and the B cell receptor signaling pathway, among others. Furthermore, only the decreased expression group consists of genes involved in heme biosynthesis, the p53 signaling pathway, and the hematopoietic cell lineage.

**Table 1:** Significant biological pathways associated with each major group of clustered gene expression profiles.

Increased Expression Group (2111 genes)	Gene List Hits	Gene Total Hits	Fisher Exact p-value
ECM-receptor interaction	26	59	3.33×10^−11^
Focal adhesion	43	161	3.43×10^−9^
Cell Communication	23	82	7.92×10^−6^
Matrix Metalloproteinases	8	17	2.36×10^−5^
Cell adhesion molecules (CAMs)	26	111	6.73×10^−5^
Wnt Signaling	8	25	6.07×10^−4^
Colorectal cancer	16	70	2.28×10^−3^
Arginine and proline metabolism	8	24	2.50×10^−3^
Adherens junction	14	62	4.76×10^−3^
Bladder cancer	9	32	4.97×10^−3^
Axon guidance	19	96	5.27×10^−3^
TGF Beta Signaling Pathway (GenMAPP)	7	28	6.66×10^−3^
Inflammatory Response Pathway	6	22	7.71×10^−3^
Alzheimer's disease	7	25	1.32×10^−2^
TGF-beta signaling pathway (KEGG)	14	70	1.43×10^−2^
Blood Clotting Cascade	3	7	1.64×10^−2^
Orphan GPCRs	3	8	2.46×10^−2^
Basal cell carcinoma	9	42	3.03×10^−2^
Melanogenesis	14	79	3.81×10^−2^
Antigen processing and presentation	11	58	4.05×10^−2^

Variable Expression Group (4373 genes)	Gene List Hits	Gene Total Hits	Fisher Exact p-value
Glycolysis and Gluconeogenesis	16	31	9.96×10^−5^
Notch signaling pathway	18	40	5.05×10^−4^
Pyruvate metabolism	14	28	5.77×10^−4^
Aminosugars metabolism	10	17	7.02×10^−4^
Natural killer cell mediated cytotoxicity	29	81	1.27×10^−3^
Fatty acid synthesis	9	16	1.79×10^−3^
Nicotinate and nicotinamide metabolism	7	11	2.57×10^−3^
Citrate cycle (TCA cycle)	10	20	3.61×10^−3^
Renal cell carcinoma	22	62	5.42×10^−3^
Pancreatic cancer	22	63	6.73×10^−3^
GnRH signaling pathway	26	81	1.16×10^−2^
Fc epsilon RI signaling pathway	22	66	1.23×10^−2^
Gap junction	25	79	1.59×10^−2^
N-Glycan biosynthesis	14	38	1.73×10^−2^
Biosynthesis of steroids	9	21	1.90×10^−2^
Carbon fixation	8	18	2.10×10^−2^
Terpenoid biosynthesis	3	4	3.09×10^−2^
B cell receptor signaling pathway	17	53	3.77×10^−2^
Calcium Channels	5	10	3.79×10^−2^
Glycosaminoglycan degradation	4	7	3.89×10^−2^
Pentose phosphate pathway	7	17	4.68×10^−2^

Decreased Expression Group (2578 genes)	Gene List Hits	Gene Total Hits	Fisher Exact p-value
Cell cycle (KEGG)	38	97	3.47×10^−13^
Cell cycle (GenMAPP)	10	19	5.39×10^−6^
Heme Biosynthesis	5	5	1.36×10^−5^
Eicosanoid Synthesis	6	12	7.49×10^−4^
p53 signaling pathway	14	54	1.90×10^−3^
DNA polymerase	8	23	2.51×10^−3^
Purine metabolism	21	100	2.92×10^−3^
Porphyrin and chlorophyll metabolism	7	19	3.23×10^−3^
One carbon pool by folate	4	9	1.25×10^−2^
Small ligand GPCRs	4	10	1.65×10^−2^
ABC transporters - General	7	26	2.11×10^−2^
Blood Clotting Cascade	3	7	3.17×10^−2^
Sphingolipid metabolism	6	23	3.71×10^−2^
Glioma	11	55	3.89×10^−2^
Hematopoietic cell lineage	13	70	4.49×10^−2^
Folate biosynthesis	4	13	4.93×10^−2^
Glycosphingolipid biosynthesis - ganglioseries	4	13	4.93×10^−2^

**Figure 5:**
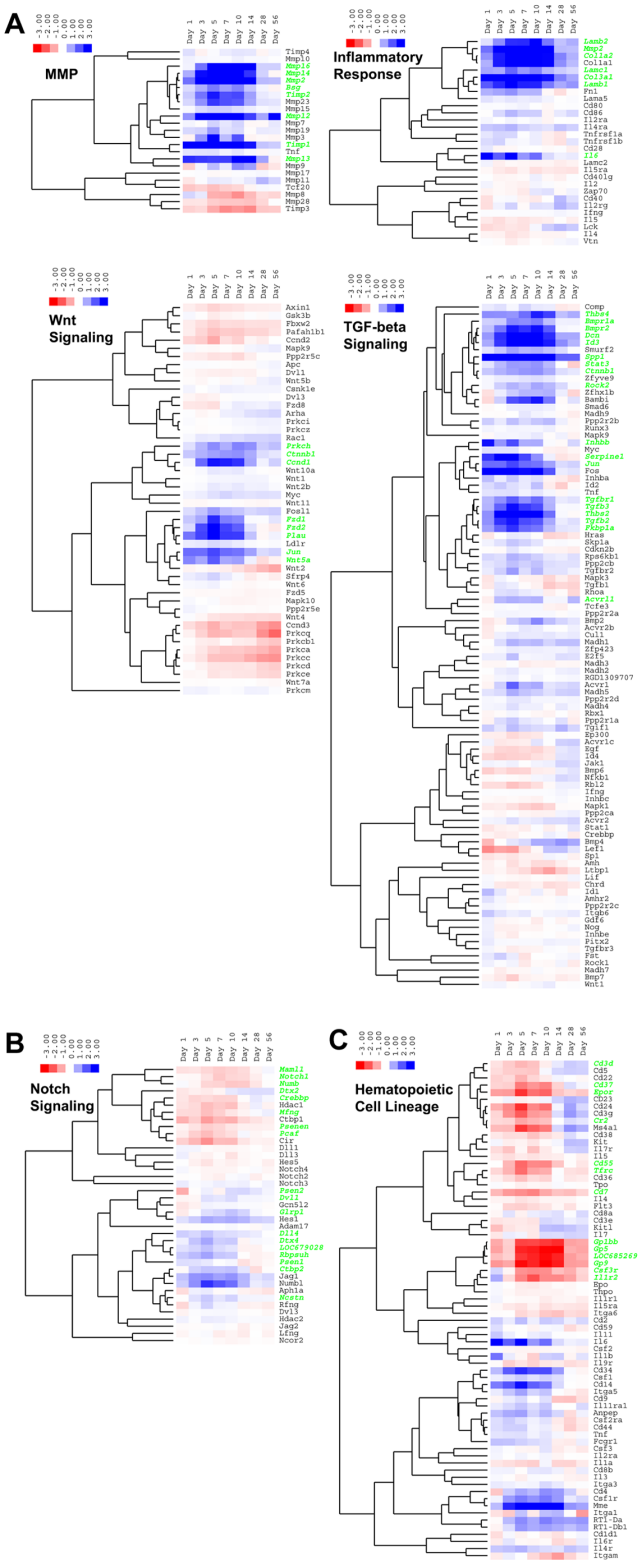
Clustered heat maps of genes involved in significant pathways identified for each major group of clustered gene expression profiles. Cluster 3.0 was used to cluster the log base 2 fold-change expression values (vs. day 0 time point) of genes associated with certain significant biological pathways of interest from the three major temporal expression groupings (increased, decreased, and variable expression). Pathways of interest from the Increased Expression Group include those for matrix metalloproteinases (MMPs) (GenMAPP), Wnt signaling (GenMAPP), TGF-β signaling (KEGG and GenMAPP), and inflammatory response pathway (GenMAPP) shown in **Figure 5A**. A pathway of interest from the Variable Expression Group is notch Signaling Pathway (KEGG) shown in **Figure 5B**, and a pathway of interest from the Decreased Expression Group is hematopoietic cell lineage (KEGG) shown in **Figure 5C**. The log base 2 fold-change ratios were clustered using hierarchical clustering with a centered correlation distance/similarity metric and average linkage clustering method. The clustered data table file was viewed in TreeView using the pixel setting contrast default of 3 and using blue and red to represent positive and negative fold-change expression values, respectively. The resulting clustered heat maps display the fold-change (vs. day 0 time-point) expression values for each gene in the rows, and for each time point in the columns, and the order of the rows is based on the result of clustering genes of similar profiles. Each heat map displays the total list of genes on the Affymetrix GeneChip® Rat Genome 230 2.0 Array that are associated with the particular pathway of interest. Genes from the initial significant gene list of 9,062 (and noted as “Gene List Hits” in Table 1) associated with each pathway are highlighted with a bold green font for their gene symbols on the right side of the heat maps.

Genes associated with significant pathways of interest from the three major temporal expression groups were hierarchically clustered based on their expression profiles and are presented as heat maps (**Figure 5**) showing fold-change values (vs. day 0 time point) of log base 2 expression values. Pathways associated with the increased expression group that are displayed as heat maps (**Figure 5A**) include matrix metalloproteinases (MMPs) (MMP), Wnt signaling pathway, TGF-β signaling pathway, and inflammatory pathway. Other pathways associated with the variable or decreased expression group that are displayed as heat maps are notch signaling pathway (**Figure 5B**) and hematopoietic cell lineage pathway (**Figure 5C**), respectively.

### Identification of significant biological pathways associated with each time point post-ablation of marrow

As opposed to significant pathways identified to be associated with the three major temporal groups based on clustering of all significant genes, significant (p<0.05) biological pathways were also identified by analyzing the expression of significant genes at each time point following bone marrow ablation. Pathway results for significant gene expressions on each day vs. day 0 are shown in **Table 2** **,** **Table 3** **, and** **Table 4**. All days except Day 56 exhibit the general pathways of ECM-receptor interaction and focal adhesion as significant in their resulting lists, with the majority of time points exhibiting other general pathways related to cell communication or cell cycle. Collectively, the inflammatory response and blood clotting cascade pathways are shown to be significant at all time points. Other pathways of interest, such as those shown in **Figure 5** are significantly expressed only at certain time points. For example, Wnt signaling and matrix metalloproteinases (MMPs) were identified to be differential pathways involved on Days 1 to Day 7, and Day 1 to Day 10, respectively, post-ablation of bone marrow. Interestingly, axon guidance and the p53 signaling pathway are found to be significantly expressed exclusively on Day 10, and the VEGF signaling pathway is identified as significant uniquely on Day 14.

**Table 2:** Significant biological pathways identified at day 1, 3, and 5 (vs. day 0) post-ablation.

Day 1 vs Day 0 (1163 genes)	Gene List Hits	Gene Total Hits	Fisher Exact p-value
ECM-receptor interaction	20	59	3.17×10^−10^
Focal adhesion	24	161	1.08×10^−4^
Cell Communication	15	82	2.42×10^−4^
Inflammatory Response Pathway	7	22	1.02×10^−3^
Glycolysis and Gluconeogenesis	8	31	2.01×10^−3^
Small cell lung cancer	11	69	5.11×10^−3^
Glycolysis/Gluconeogenesis	8	42	5.50×10^−3^
Hematopoietic cell lineage	11	70	5.71×10^−3^
Cell adhesion molecules (CAMs)	15	111	5.87×10^−3^
Cytokine-cytokine receptor interaction	16	129	1.03×10^−2^
Renal cell carcinoma	9	62	1.98×10^−2^
Nucleotide sugars metabolism	2	4	2.42×10^−2^
ABC transporters - General	5	26	2.58×10^−2^
Prion disease	3	11	3.21×10^−2^
Porphyrin and chlorophyll metabolism	4	19	3.34×10^−2^
Matrix Metalloproteinases	4	17	4.15×10^−2^
Wnt Signaling	5	25	4.40×10^−2^
Ether lipid metabolism	4	21	4.65×10^−2^

Day 3 vs Day 0 (2670 genes)	Gene List Hits	Gene Total Hits	Fisher Exact p-value
ECM-receptor interaction	26	59	5.97×10^−9^
Focal adhesion	46	161	1.63×10^−7^
Matrix Metalloproteinases	9	17	4.54×10^−5^
Cell Communication	24	82	1.14×10^−4^
Heme Biosynthesis	4	5	9.77×10^−4^
Cell adhesion molecules (CAMs)	27	111	1.18×10^−3^
Wnt Signaling	9	25	1.64×10^−3^
Arginine and proline metabolism	9	24	2.69×10^−3^
Inflammatory Response Pathway	8	22	2.84×10^−3^
Small cell lung cancer	18	69	3.43×10^−3^
Cell cycle	23	97	3.82×10^−3^
Colorectal cancer	18	70	4.06×10^−3^
Adherens junction	16	62	6.36×10^−3^
Porphyrin and chlorophyll metabolism	7	19	8.96×10^−3^
Aminosugars metabolism	6	17	1.93×10^−2^
Bladder cancer	9	32	2.15×10^−2^
Carbon fixation	6	18	2.57×10^−2^
Glycolysis and Gluconeogenesis	8	31	2.73×10^−2^
Glycan structures - biosynthesis 2	11	46	3.76×10^−2^
Alzheimer's disease	7	25	4.18×10^−2^
Hematopoietic cell lineage	15	70	4.22×10^−2^
Blood Clotting Cascade	3	7	4.37×10^−2^
Glycolysis/Gluconeogenesis	10	42	4.76××10^−2^

Day 5 vs Day 0 (5823 genes)	Gene List Hits	Gene Total Hits	Fisher Exact p-value
ECM-receptor interaction	34	59	5.52×10^−7^
Cell cycle	44	97	6.09×10^−5^
Focal adhesion	65	161	1.06×10^−4^
Cell cycle	12	19	4.12×10^−4^
Heme Biosynthesis	5	5	9.54×10^−4^
Blood Clotting Cascade	6	7	1.30×10^−3^
Matrix Metalloproteinases	10	17	2.83×10^−3^
Arginine and proline metabolism	13	24	4.24×10^−3^
Aminosugars metabolism	10	17	5.57×10^−3^
p53 signaling pathway	23	54	9.05×10^−3^
Carbon fixation	10	18	9.54×10^−3^
Gap junction	31	79	1.11×10^−2^
Glioma	23	55	1.17×10^−2^
Adherens junction	25	62	1.48×10^−2^
Porphyrin and chlorophyll metabolism	10	19	1.53×10^−2^
Colorectal cancer	27	70	2.18×10^−2^
Wnt Signaling	11	25	2.80×10^−2^
Hematopoietic cell lineage	26	70	3.91×10^−2^

**Table 3:** Significant biological pathways identified at day 7 and 10 (vs. day 0) post-ablation.

Day 7 vs Day 0 (2205 genes)	Gene List Hits	Gene Total Hits	Fisher Exact p-value
ECM-receptor interaction	32	59	4.39×10^−16^
Focal adhesion	45	161	2.13×10^−9^
Cell Communication	24	82	6.43×10^−6^
Matrix Metalloproteinases	8	17	2.04×10^−4^
Blood Clotting Cascade	5	7	2.95×10^−4^
Cell adhesion molecules (CAMs)	24	111	1.15×10^−3^
Complement and coagulation cascades	14	53	1.75×10^−3^
Wnt Signaling	8	25	4.22×10^−3^
Colorectal cancer	16	70	4.23×10^−3^
Gap junction	17	79	6.23×10^−3^
Eicosanoid Synthesis	5	12	7.00×10^−3^
Bladder cancer	9	32	7.41×10^−3^
Inflammatory Response Pathway	7	22	7.80×10^−3^
Melanoma	14	64	1.09×10^−2^
Small cell lung cancer	14	69	2.07×10^−2^
Glycosphingolipid biosynthesis - neo-lactoseries	5	15	2.11×10^−2^
Adherens junction	12	62	4.32×10^−2^

Day 10 vs Day 0 (2190 genes)	Gene List Hits	Gene Total Hits	Fisher Exact p-value
ECM-receptor interaction	27	59	2.43×10^−12^
Focal adhesion	44	161	4.23×10^−10^
Matrix Metalloproteinases	9	17	3.05×10^−6^
Cell adhesion molecules (CAMs)	24	111	3.23×10^−4^
Cell Communication	19	82	5.51×10^−4^
Axon guidance	21	96	6.50×10^−4^
Cell cycle	20	97	1.90×10^−3^
Glycosaminoglycan degradation	4	7	3.16×10^−3^
Bladder cancer	9	32	4.19×10^−3^
p53 signaling pathway	12	54	8.18×10^−3^
Adherens junction	13	62	9.97×10^−3^
Gap junction	15	79	1.47×10^−2^
Blood Clotting Cascade	3	7	1.90×10^−2^
Glioma	11	55	2.41×10^−2^
Colorectal cancer	13	70	2.65×10^−2^
Leukocyte transendothelial migration	17	100	2.76×10^−2^
Orphan GPCRs	3	8	2.84×10^−2^
Melanoma	12	64	3.03×10^−2^
Inflammatory Response Pathway	5	22	4.04×10^−2^

**Table 4:** Significant biological pathways identified at day 14, 28, and 56 (vs. day 0) post-ablation.

Day 14 vs Day 0 (459 genes)	Gene List Hits	Gene Total Hits	Fisher Exact p-value
Focal adhesion	18	161	6.75×10^−7^
ECM-receptor interaction	9	59	4.73×10^−5^
Long-term depression	8	68	7.99×10^−4^
Thyroid cancer	5	27	1.03×10^−3^
Blood Clotting Cascade	3	7	1.66×10^−3^
Glycan structures - degradation	3	14	7.43×10^−3^
Leukocyte transendothelial migration	8	100	9.20×10^−3^
Adherens junction	6	62	9.72×10^−3^
Endometrial cancer	5	45	1.02×10^−2^
Bladder cancer	4	32	1.42×10^−2^
Glycosaminoglycan degradation	2	7	1.69×10^−2^
MAPK Cascade	3	15	1.75×10^−2^
Tight junction	8	115	2.03×10^−2^
ErbB signaling pathway	6	75	2.34×10^−2^
Dorso-ventral axis formation	3	22	2.65×10^−2^
Glycosphingolipid biosynthesis - globoseries	2	9	2.78×10^−2^
N-Glycan degradation	2	9	2.78×10^−2^
Melanogenesis	6	79	2.93×10^−2^
GnRH signaling pathway	6	81	3.27×10^−2^
VEGF signaling pathway	5	61	3.42×10^−2^
Cell Communication	6	82	3.44×10^−2^
Renal cell carcinoma	5	62	3.64×10^−2^
Nicotinate and nicotinamide metabolism	2	11	4.09×10^−2^
alpha-Linolenic acid metabolism	2	11	4.09×10^−2^
Fc epsilon RI signaling pathway	5	66	4.58×10^−2^

Day 28 vs Day 0 (532 genes)	Gene List Hits	Gene Total Hits	Fisher Exact p-value
ECM-receptor interaction	13	59	2.17×10^−8^
Focal adhesion	16	161	3.89×10^−5^
Cell Communication	10	82	2.38×10^−4^
Hematopoietic cell lineage	9	70	3.32×10^−4^
Inflammatory Response Pathway	4	22	8.20×10^−3^
Fatty acid biosynthesis	2	5	9.67×10^−3^
Glyoxylate and dicarboxylate metabolism	2	7	1.95×10^−2^
Fatty acid synthesis	3	16	2.10×10^−2^
Reductive carboxylate cycle (CO2 fixation)	2	8	2.54×10^−2^
Blood Clotting Cascade	2	7	2.72×10^−2^

Day 56 vs Day 0 (100 genes)	Gene List Hits	Gene Total Hits	Fisher Exact p-value
Glycosphingolipid biosynthesis - lactoseries	1	7	4.12×10^−2^
Antigen processing and presentation	2	58	4.60×10^−2^
Cholesterol biosynthesis	1	10	4.61×10^−2^
Sulfur metabolism	1	8	4.70×10^−2^

### Identification of significant transcription factors expressed at each time point post-ablation of marrow

**Figure 6:**
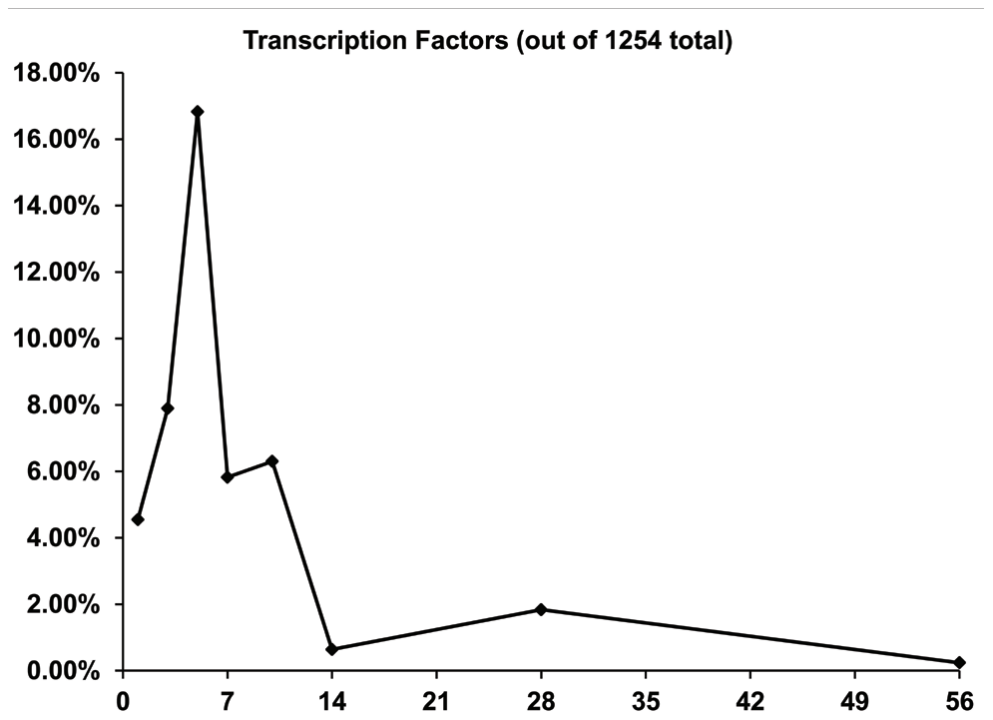
Percentage distribution of significant transcription factors expressed on each time point (each day vs. day 0) post-ablation. The total number of probe sets for transcription factors known to be present on the Affymetrix GeneChip® Rat Genome 230 2.0 Array was determined to be 1,254. Significant probe set lists for each time point (each day vs. day 0) were compared with the transcription factors list, and the number of significant transcription factors expressed for each time point was found. The percentage of the transcription factors expressed was calculated and graphed for each time point.

The XY-scatter plot graph (**Figure 6**) displays the percentage distribution of significant transcription factors expressed at each time point, and **Table S3** provides the list of Affymetrix GeneChip® Rat Genome 230 2.0 Array probe set ID and transcription factor names for each time point. The complete list of probe sets for transcription factors present on the Affymetrix GeneChip ® Rat Genome 230 2.0 Array was found to be 1,254. Comparing the significant probe set lists for each time point post-ablation (each day vs. day 0) with the master list of known transcription factors, the number of transcription factors (TFs) expressed at each time point was determined to be: 42 TFs at Day 1, 99 TFs at Day 3, 211 TFs on Day 5, 73 TFs at Day 7, 79 TFs at Day 10, 8 TFs at Day 14, 23 TFs at Day 28, and 3 TFs on Day 56. Therefore, the number of significant transcription factors markedly peaked at 5 days post-ablation, where there was more than twice the number of transcription factors differentially expressed compared to any other time point. It is shown in the graph in Figure 6 that the percentage of transcription factors significantly expressed on Day 5 out of the total number of transcription factors (1,254) on the Affymetrix GeneChip® Rat Genome 230 2.0 Array was roughly 17%.

## Discussion

This study was the first to identify significant biological processes and pathways from genome-wide transcriptional analysis during all phases of intramembranous bone regeneration following marrow ablation in a rat model. We used an oligonucelotide microarray technology, specifically fabricated by Affymetrix for Rat Genome 230 2.0, and used univariate F-test to determine a list of 9,062 gene probe sets significantly differentially expressed over all time points, which is roughly 30% of the 31,042 gene probe sets present on the total microarray. The time points of days 1, 3, 5, 7, 10, 14, 28, and 56 days post-ablation of marrow were selected based on knowledge of the complete timeline of all general bone healing processes involving phases of inflammation, repair and new bone formation, and remodeling. This timeline is confirmed by the data presented, implicating that most expression profiles return to baseline at 56 days, as shown by the average expression profiles (**Figure 3**). A Bayesian model-based clustering method was used to determine major temporal expression groups based on gene expression profiles. The percentage distribution of significant biological processes (**Figure 4** and **Table S2A–D**) and the differential biological/signaling pathways (**Table 1**) identified for increased, decreased, and variable expression groups serve as a genome-wide overview of the main biological functions that occur during marrow ablation-induced intramembranous bone regeneration in the rat femur. Additionally, identification of biological pathways (**Table 2** **,** **Table 3** **,** and **Table 4**) and transcription factors (**Figure 6** and **Table S3**) significantly expressed at each time point post-ablation provides important insight into the regulation of intramembranous bone regeneration in a complete temporal context.

Each of the phases of new bone formation identified in the histologic analysis generally progress in an “outside-in” fashion toward the core of the initial clot matrix. Thus, even though distinct days can be assigned to a given phase, because of the spatial and temporal heterogeneity inherent in the regenerative process, almost all stages can be identified in some, albeit limited, regions of the ablation site in samples recovered between days 7 and 10. This heterogeneity implies that peaks of activity occur concurrently in different locations and that critical events and phenotypic transformations reflected in gene expression at these transformative sites may occupy small footprints in the overall expression profiles. Alignment of gene expression patterns with the phases described above (see Results section) in their spatial context may eventually help identify genes that are critical to bone regeneration but appear at low levels within the more dominant spatial domains at any particular time.

Previous studies of gene expression using marrow ablation models selectively investigated individual or very limited number of osteogenesis-associated cytokines or growth factors which are described and summarized in previous publications by our group[2], [19]. Results determined by the present study complement conclusions highlighted in those previous publications, in that intramembranous bone regeneration generally relies upon the control of gene expression for inflammatory cytokines, osteogenic growth factors, transcription factors, matrix proteins and proteinases, and bone remodeling markers.

Focusing on the most differentially expressed genes in Clusters 8, 1, and 7 in **Figure 3A** (**Increased Expression**) and **Table S1**, several osteoblastic- and osteogenesis-associated genes of interest[2] are identified including osteopontin (SPP1 in Cluster 8 [25353]), dentin matrix protein 1 (Dmp1 in Cluster 8 [25312]), osteonectin (also known as Sparc in Cluster 1 [24791]), osteocalcin (also known as Bglap in Cluster 7 [25295]), collagen type I (Col1a1 [29393] and Col1a2 [84352]) in Cluster 1), alkaline phosphatase (Alp1 in Cluster 1 [25586]), and periostin (Postn in Cluster 8 [361945]). It is known that periostin has a functional role as a matricellular protein in a variety of tissue remodeling and wound repair situations[32], and it has been recently confirmed that periostin is a novel marker of intramembranous ossification, but not endochondral ossification, and exhibits upregulated expression in the fibrous component of fibrous dysplasia, due to its prominent influence on collagen fibrillogenesis[33]. Many of the aforementioned osteoblastic-associated genes exhibit peak upregulation at day 5 or Day 10, which subsequently and, respectively, result in bone formation observed in the histology tissue by Days 10 and 14 (**Figure 2**).

Clusters 8, 1, and 7, in **Figure 3A** (**Increased Expression**) and **Table S1**, also include a number of osteoclastic- and bone matrix remodeling-associated genes of interest[2], including TIMP1 (Cluster 8 [116510]), TIMP2 (Cluster 1 [29543]), MMP2 (Cluster 8 [81686), MMP12 (Cluster 8 [117033]), MMP13 (Cluster 1 [171052]), MMP14 (also known as MT1-MMP in Cluster 1 [81707]), MMP16 (Cluster 1 [65205]), MMP23 (Cluster 1 [94339]), cathepsin K (Ctsk in Cluster 8 [29175]), TRAP5 (Acp5 in Cluster 1 [25732]), osteoprotegerin (Tnfrsf11b in Cluster 1 [25341]), PTHr1 (Cluster 1 [56813]), and RANK (Tnfrsf11a in Cluster 7 [498206]). The aforementioned osteoclastic-associated genes exhibit peak upregulation at day 10, which subsequently result in bone resorption and bone remodeling observed histologically in the tissue by Day 14 and 28 (**Figure 2**).

No evidence of chondrogenesis was observed histologically in the tissue at any time point, and two major chondrogenic marker genes collagen type II (Col2) and transcription factor Sox9 were not identified as significantly expressed in our model. Therefore, true chondrogenesis was not identified in this model. However, other chondrocytic-related marker genes were found within the significantly expressed genes in **Table S1** and in **Figure 3A** and **Figure 3B**, which include aggrecan (Acan in Cluster 1 [58968]), versican (Vcan in Clusters 1 and 6 [114122]), and syndecan 3 (Sdc3 in Cluster 6 [116673]), where they exhibit peak upregulation at day 5 followed by a rather steep decline to return to baseline by day 14. This is suggestive of a transient existence of chondrogenesis. This observation is similar to the one reported by Nah *et al* [34] on the “covert chondrogenic phase” in their study of intramembranous bone formation in chick embryonic frontal bone and sternum.

Adipocyte marker genes that are also found to be significantly expressed (**Table S1**, **Figure 3B** and **Figure 3C**) include Pparg (in Cluster 2 [25664]), adiponectin (Adipoq in Cluster 2 [246253], Adipor1 in Cluster 4 [289036], Adipor2 in Cluster 5 [312670]), and Leptin receptor (Lepr in Clusters 2 and 4 [24536]). Interestingly, all the expression profiles of Clusters 2, 4, and 5 which contain adipocytic markers display a downregulated expression profile during day 5 until day 10, which contrasts the expression profiles of Clusters 8, 1, and 7 which contain osteoblastic-associated marker genes and which show peak upregulation during day 5 to day 10. In other words, adipocytic-associated genes are downregulated while osteoblastic-associated genes are upregulated. Adipocytic-assocatied genes in Clusters 4 and 5 return to baseline by Day 28, leading to restoration of the fatty marrow, while adipocyte markers genes contained within Cluster 2 become upregulated at Day 14 and remain so until day 56.

One of the major pathways identified in our model to be significantly expressed in the increased expression group (**Table 1**), was the molecular function network of matrix metalloproteinases (MMPs), which was specifically determined to be significantly expressed at 1, 3, 5, 7, and 10 days post-ablation (**Table 2** and **Table 3**). Eight MMP-specific genes with significant increased expression that were identified using the GenMAPP database were Bsg, Timp 1, Timp 2, MMP2 (gelatinase A or type IV collagenase), MMP12 (metalloelastase), MMP13 (collagenase), MMP14 (membrane-type 1 MMP), and MMP16 (membrane-type 3 MMP), and these are highlighted in the heat map for MMP genes in **Figure 5A**. It can be seen that the expression values of MMP2, MMP14, TIMP2, and MMP16 are hierarchically clustered together. This result is expected since MMP14 (also known as MT1-MMP) and TIMP-2 are specifically involved in the cell surface mechanism to activate proMMP-2[35], [36], that essentially contributes to the promoting the invasive capacity of tumor cells[35] and mesenchymal stem cells[37], and it is also known that MMP16 (MT3-MMP) activates MMP2 by cleavage of proMMP-2[38]. From the results of the temporal expression profile clustering, the most up-regulated genes were TIMP1, MMP2, and MMP12 (Cluster 8 in **Figure 3A** and **Table S1**). Other MMPs that exhibited significant increased expression were TIMP2, MMP13, MMP14, and MMP16 (Cluster 1 in **Figure 3A** and **Table S1**). MMP13, a known collagenase, exhibited sustained strong induction in our model for up to 14 days post-ablation, and is known to have important functions in bone formation and remodeling. It has previously been reported that MMP13 is specifically required to cleave collagen type II and aggrecan during the transition from cartilage to bone at the growth plate during long bone development, in addition to mediating initial and continual remodeling of trabeculae during ossification[39], [40]. This knowledge correlates with the relationship of MMP13 and Aggrecan gene expression observed in the data from our model. Aggrecan was found as a significantly upregulated gene in our model (Cluster 1 in **Figure 3A** and **Table S1**) with its peak expression at the day 5 and 7 time points. MMP13 reached peak expression on day 10 and 14, during which a significant decline towards baseline was seen for Aggrecan expression. It has also been recently reported, using non-stabilized or stabilized fracture models in mice, that MMP13 is required for both endochondral and intramembranous ossification during bone repair, likely for initial degradation of ECM prior to the invasion of blood vessels and osteoclasts[41]. These interesting findings implicate that some extent of transient chondrogenesis is occurring in our model in addition to the predominant intramembranous bone regeneration induced by bone marrow ablation, even though cartilage were not histologically evident. Furthermore, our previous study demonstrated that a biomimetic polymer hydrogel with a semi-interpenetrating network that incorporates an MMP13 degradable crosslinker peptide and an integrin-binding peptide (containing the RGD domain) implanted in a rat femoral ablation model leads to significantly enhanced bone regeneration, likely due to enhanced osteoblast migration and proliferation in such constructs[18]. Further studies involving tailored design of biomaterials could benefit from the knowledge presented here regarding MMP-specific genes to target particular MMP-related peptide sequences for certain types of tissue regeneration.

Another key pathway identified to be significantly expressed in the increased expression group (**Table 1**) was the cellular process pathway of Wnt signaling, which was found to be significantly expressed at the study timepoints of 1, 3, 5, and 7 days post-ablation. A study by Kim *et al.* used a model where bone regeneration in a 1.0-mm monocortical tibial defect occurs exclusively through intramembranous ossification, and they reported that Wnt signaling is upregulated at the injury site, thereby prompting marrow-derived osteoprogenitor cells to proliferate and mediating subsequent osteoblast differentiation[42]. In our model, Wnt signaling related genes for Wnt5a [64566], Fzd1[58868], Fzd2 [64512], Prkch [81749], and Ctnnb1 (β-catenin [84353]), as well as transcriptional activation genes for Ccnd1 [58919], Jun [24516], and Plau [25619], were found to be significantly upregulated as identified by the GenMAPP database, and are highlighted in the heatmap for Wnt signaling genes in **Figure 5A** (and are also found in Cluster 7 in **Figure 3A** and **Table S1**). In the previously mentioned study by *Kim et al.*, Wnt5a was also identified as significantly expressed in all domains of the injury site in their primarily intramembranous ossification model, which included the osteocytes, endosteum, bone marrow, growth plate, and the periosteum, which was the only domain adjacent to the injury site where there was an endochondral ossification response[42].

The present results with marrow ablation-induced intramembranous bone regeneration share similarities with two other microarray studies using rat closed fracture models, which also identified genes for Wnt5a, Frizzled 2, and β-catenin[1], [43]. In the study by Hadjiargyrou *et al.*, the identified temporal expression for Wnt5a was interestingly reported to increase at day 3 post-fracture, then decline at days 5–7, then return to an increased expression at 10–14 days, and finally drop back down to baseline levels by 21 days[1]. A similar but more early and transient temporal expression profile for Wnt5a was found in our model, with an increase at day 1 post-ablation, then a decrease on day 3, then a marked increase at day 5, then another decrease at days 7 and 10, followed by a further decrease towards baseline by 14 days. Wnt5a is known as a non-canonical Wnt ligand that signals independent of β-catenin through the Wnt/Ca^2+^ pathway, which not only regulates Ca^2+^ flux and Ca^2+^-sensitive protein kinases and transcription factors, but can inhibit the canonical Wnt pathway by promoting degradation of β-catenin[44]. Specific mechanisms involved with the regulation of bone regeneration by non-canonical Wnt pathway are still relatively unknown, but several studies have shown evidence supporting the role of Wnt5a in the early stages of fracture repair, namely inflammation and chondrogenesis[1], [44]. Additionally, there is other evidence that Wnt5a induces osteoblastogenesis by reducing PPARγ-induced adipogenesis in bone marrow mesenchymal stem cells[45]. Considering this knowledge of Wnt5A, in addition to the information previously discussed regarding MMP13, it is possible that early and transient chondrogenesis may be occurring in our predominantly intramembranous regeneration model induced by marrow ablation. Altogether, these intriguing findings from our study and many other reports implicate that Wnt signaling in bone repair models, fracture or marrow ablation, is highly intricate and currently not well understood, evidently involving the activation of both canonical and non-canonical Wnt signaling pathways[44], [46].

It is generally known during the early bone repair phase that mesenchymal stem cells (MSCs) migrate, proliferate, and undergo differentiation towards the osteoblastic lineage. It is of interest to further investigate signaling pathways and genes expressed by MSCs during this phase for overall enhancement and acceleration of bone regeneration following injury and surgery, and the temporal transcriptional profiling data results presented here are expected to aid in that research. Signaling pathways known to be in involved in the self-renewal and osteogenic differentiation of MSCs are Wnt signaling, BMP/TGF-β signaling, and notch signaling[47], which are pathways identified in this study to be significantly expressed within the increased or variable expression groups, respectively. There is evidence that MSCs express Wnt2, Wnt4, Wnt5a, Wnt11, Wnt16, Fzd2, Fzd3, Fzd4, Fzd5, Fzd6, and Dkk1[48], and that canonical Wnt3a increases levels of β-catenin and the proliferation rate while noncanonical Wnt5a impedes the process of chondrogenesis[47]. Indeed, there is cross-talk between canonical and noncanonical Wnt signaling in MSCs, as canonical Wnt3a suppresses Wnt5a to maintain MSC in an undifferentiated and self-renewing state, while noncanonical Wnt5a inhibits Wnt3a in order to mediate enhancement of osteogenic differentiation of MSCs[49], [50]. Specifically, Wnt5a suppresses β-catenin/TCF signaling to decrease the level of cyclin D1 and proliferation rate of MSCs[49], [51].

The notch signaling pathway is thought to be involved in osteoblastogenesis and skeletogenesis but its particular role is not well understood[47]. It has been reported that within the bone marrow, notch signaling suppresses osteoblastic differentiation and the Wnt/β-catenin signaling pathway generally acts to maintain a pool of proliferating mesenchymal progenitors[52], [53]. Although not specifically identified as significant by the KEGG database through DAVID EASE, genes involved in the notch Signaling Pathway and differentially expressed over all time points in our model, include Hes1 [29577], Jag1 [29146], and Numbl [292732]. It was recently confirmed that Hes1 plays an important role in mediating the inhibition of osteoblastogenesis and the Wnt/β-catenin pathway by intracellular domains of notch signaling, likely by preventing the interaction of β-catenin with the transcriptional co-repressor Groucho/TLE and LEF-1[53]. The notch ligand gene Jag1 is known as an evolutionarily conserved target of the canonical Wnt signaling pathway and is a key molecule for induction of self-renewal and maintenance of homeostasis of stem and progenitor cells[54], while Numb and Numbl induce differentiation by inhibiting notch signaling in progenitor cells[55].

Regarding the TGF-β signaling pathway, many significantly expressed genes were identified by the KEGG or GenMAPP databases and are shown as three major clusters in the TGF-β signaling pathway heat map in **Figure 5A**. One notable cluster includes Thbs4 [29220], Bmpr1a [81507], Bmpr2 [140590], Dcn [29139], Id3 [25585], Spp1 (osteopontin [25353]), Stat3 [25125], Ctnnb1 [84353], and Rock2 [25537]. Interestingly, it has been shown with mouse embryonic cells that Id proteins, such as Id3, in conjunction with STAT3 sustain stem cell self-renewal and inhibit differentiation[56]. Id3 is a significant transcription factor in our data and can be seen listed for Days 3, 5, 7, and 10 post-ablation in **Table S3**. Another cluster of TGF-β related genes consists of Inhbb [25196], Serpine1 [24617], and Jun [24516], and a third major cluster contains Tgfbr1 [29591], Tgfb3 [25717], Thbs2 [292406], Tgfb2 [81809], and Fkbp1a [25639]. It is well accepted that BMP signaling has an important role in osteoblastogenesis, and TGF-βs can have both positive and negative effects on osteoblast differentiation. It has been shown that bone formation is induced by injecting TGF-β into periosteum, but on the other hand, an osteoporotic phenotype resulted with an overexpression of TGF-β2[47]. Indeed, it has been shown previously by our group and others that intramembranous bone formation associated with implant fixation is enhanced following local delivery of TGF-β[11], [57]–[59]. Interestingly, in our data Thbs2, which is clustered with TGF-β2, is highly expressed from day 3 to day 10 post-ablation, and has been described as an autocrine inhibitor of proliferation secreted by MSCs[60] and an important regulator, along with osteonectin (also known as Sparc), of the osteoblast lineage and bone remodeling[61].

There are a few recent reports regarding genome-wide transcriptional profiling of mouse and human MSCs and the identification of differentially expressed genes and pathways[62], [63]. Specifically it was reported that transcripts uniquely expressed by murine bone marrow-derived MSCs are mostly transcription factors and genes downstream of the Wnt signaling pathway and also MSCs exhibit a unique therapeutic effect on T cells by preventing their proliferation and supporting their survival[62]. Another study performed microarray-based genome-wide differential gene expression analysis to identify the 20 most significantly expressed genes in human bone marrow-derived MSCs, including several we have identified as differentially expressed in our model of intramembranous bone regeneration such as Periostin (Postn [361945]), Col3a1 [84032], Col6a3 [367313], Cthrc1 [282836], Lysyl oxidase [24914] (Cluster 8 in **Table S1**), and Col1a1 [29393], Ctgf [64032], Col1a2 [84352], Serpine1 [24617], Kdelr3 [315131] (Cluster 1 in **Table S1**)[63].

There are many noteworthy similarities and differences of the genome-wide transcriptional analysis results presented here for intramembranous bone regeneration induced by a marrow ablation model with results previously reported by Bais *et al.* regarding the endochondral bone formation process in fracture healing [20]. In their paper, it was reported that a little over one-half of the genes expressed in the mouse genome were differentially regulated during fracture healing[20]. Our data indicates that roughly one-third of the genes expressed on the Affymetrix GeneChip® Rat Genome 230 2.0 Array were differentially expressed during intramembranous bone regeneration and healing following marrow ablation. Interestingly, many commonalities can be seen when comparing gene ontology/biological process analysis of the three major temporal expression groups (up, variable, and down) from Bais *et al.* with the results presented here. Specifically, the many biological process that share similar percentage distributions for the up or increased expression group identified in both studies are cell adhesion, cell cycle, cytoskeleton/actin, development, immune response, ion transport, many aspects of metabolism, motility, neurogenesis, a variety of cellular signaling including Wnt (see **Table S2A–D** from this study and Table S1 from Bais *et al.*), and vasculogenesis[20]. Marked differences include that a greater percentage distribution (7.7%) for skeletogenesis is found from our increased expression group data for intramembranous bone regeneration model compared to a much lower value (1%) for skeletogenesis shown in the increased expression group in Bais *et al*. which uses a fracture healing model[20]. Other similarities and differences can be observed by comparing and contrasting the biological process percentage distribution for the variable (**Figure 4B**) and decreased (**Figure 4C**) expression groups from this study with the variable and down groups in Bais *et al.* [20].

More similarities exist with our results of significant biological pathways identified (from KEGG and GenMAPP databases) for each of the three major temporal groups (**Table 1**) when compared to pathways identified (by KEGG database) for the three major temporal groups in Bais *et al.* [20]. The increased expression groups in both studies showed significant association with ECM-receptor interaction, focal adhesion, axon guidance, focal adhesion, TGF-β signaling, cell communication, adherens junction, basal cell carcinoma, and Wnt signaling. The same pathways identified for variable group and the variable group in Bais *et al.* include B cell receptor signaling pathway and natural killer cell mediated cytotoxicity, and the same pathways identified for the decreased expression groups in both studies include cell cycle (negative regulators), hematopoietic cell lineage, ABC transporters (general), and porphyrin and chlorophyll metabolism[20]. Comparing these results from our intramembranous bone regeneration model and previously reported results from a fracture healing model implicate many similarities regarding significant biological processes and pathways during bone repair.

Given the result from this study that roughly 30% of the rat genome was significantly expressed during all time points of marrow ablation-induced intramembranous bone regeneration, it is of interest to discuss what fraction of the genome is involved in other forms of wound and tissue repair. Similar to bone, the liver has the ability to regenerate and repair with no resulting scar tissue. A genome-wide expression study using a rat liver regeneration model, in which a partial hepatectomy was performed recently reported that approximately 5.4% of the rat genome was differentially regulated during rat liver regeneration[64]. Similar to the regeneration of bone, liver regeneration involves a coordinated cascades of biological events, although our data confirms that intramembranous bone regeneration involves a much greater number of significantly activated/expressed genes compared to liver regeneration. A very recent publication using a human genome-wide microarray analysis with a cutaneous wound healing model of thermally injured skin determined that approximately 4.4% of the human genome was significantly expressed during a 18-day healing period[65]. It is known that unlike bone repair, skin repair results in scarring. Interestingly in their study, osteopontin (also known as SPP1), was highly up-regulated in the first 17 days and was significantly expressed in our intramembranous bone regeneration model (see Cluster 8 in **Figure 3** and **Table S1** and the heatmap for TGF-beta signaling genes in **Figure 5A**). The significant expression of osteopontin in wound healing supports the knowledge that it is has a broad yet critical role in injury site extracellular microenvironments containing highly proliferative cells and undergoing rapid remodeling. Another study using a rat genome-wide microarray analysis with an injured spinal cord model found that during a 90 day post-injury period, approximately 15% of the rat genome was significantly expressed[66]. It was also noted that the overall expression profiles of significantly increased tissue repair genes and the timing and sequence of post-spinal cord injury gene expression resemble the phases known for cutaneous wound healing[66]. The remarkable ability of bone to repair, such as our model of intramembranous bone regeneration, appears to involve a much larger set of significant genes comprising a substantially greater percentage of the genome than other forms of wound repair.

## Supporting Information

Table S1 Tabular summary of the significantly expressed genes of intramembranous bone regeneration across the time points of this study. Expressed genes sets are arranged by the numeric cluster assigned by the CAGED analysis (Clusters 1 through 8). Gene symbol, gene name, probe set ID on the Affymetrix GeneChip® Rat Genome 230 2.0 Array, RGD Accessions, Entrez Gene ID (*Rattus norvegicus*), and Unigene clusters (*Rattus norvegicus*) are denoted. For each gene probe set, log base 2 expression values for each time point (Day 0, 1, 3, 5, 7, 10, 14, 28, and 56) are presented, as well as the fold-change expression values for each day vs. day 0.
(3.09 MB XLSX)

Table S2 Identified significant (p<0.05) biological process ontologies for clustered gene expressions in each of the three major temporal groups during intramembranous bone regeneration. Table S2A presents the major categories of biological processes and their percentage distributions, which were determined by consolidating overlapping or related individual subcategories, and are presented in the pie graphs in Figure 4 for each of the three major temporal expression groups (increased, variable, and decreased). Table S2B, S2C, and S2D present all the individual subcategory terms of biological process ontology results, the p-value for each, and major category that each was grouped into for the increased expression group, variable expression group, and decreased expression group, respectively.
(0.05 MB XLSX)

Table S3 Significant gene probe sets known to be transcription factors expressed on each time point post-ablation (each day vs. day 0) that were determined and presented in Figure 6. For the transcription list on each time point, the Affymetrix probe set ID, gene symbol, and p-value are provided, and the genes are listed in the order of p-value significance.
(0.03 MB XLSX)

## References

[1] Hadjiargyrou M, Lombardo F, Zhao S, Ahrens W, Joo J (2002). Transcriptional profiling of bone regeneration. Insight into the molecular complexity of wound repair.. J Biol Chem.

[2] Kuroda S, Virdi AS, Dai Y, Shott S, Sumner DR (2005). Patterns and localization of gene expression during intramembranous bone regeneration in the rat femoral marrow ablation model.. Calcif Tissue Int.

[3] Gerstenfeld LC, Cullinane DM, Barnes GL, Graves DT, Einhorn TA (2003). Fracture healing as a post-natal developmental process: molecular, spatial, and temporal aspects of its regulation.. J Cell Biochem.

[4] Amsel S, Maniatis A, Tavassoli M, Crosby WH (1969). The significance of intramedullary cancellous bone formation in the repair of bone marrow tissue.. Anat Rec.

[5] Patt HM, Maloney MA (1975). Bone marrow regeneration after local injury: a review.. Exp Hemat.

[6] Ishizaka M, Tanizawa T, Sofue M, Dohmae Y, Endo N (1996). Bone particles disturb new bone formation on the interface of the titanium implant after reaming of the marrow cavity.. Bone.

[7] Hara T, Hayashi K, Nakashima Y, Kanemaru T, Iwamoto Y (1999). The effect of hydroxyapatite coating on the bonding of bone to titanium implants in the femora of ovariectomised rats.. J Bone Joint Surg.

[8] Schmidmaier G, Wildemann B, Schwabe P, Stange R, Hoffmann J (2002). A new electrochemically graded hydroxyapatite coating for osteosynthetic implants promotes implant osteointegration in a rat model.. J Biomed Mater Res.

[9] Kuroda S, Virdi AS, Li P, Healy KE, Sumner DR (2004). A low temperature biomimetic calcium phosphate surface enhances early implant fixation in a rat model.. J Biomed Mater Res.

[10] De Ranieri A, Virdi AS, Kuroda S, Healy KE, Sumner DR (2005). Saline irrigation does not affect bone formation or implant fixation strength in a rat model.. Journal of Biomedical Materials Research Part B: Applied Biomaterials.

[11] De Ranieri A, Virdi AS, Kuroda S, Shott S, Leven RM (2005). Local application of rhTGF-β2 enhances peri-implant bone volume and bone-implant contact in a rat model.. Bone.

[12] Sena K, Sumner DR, Virdi AS (2010). Effect of recombinant human transforming growth factor-beta2 dose on bone formation in rat femur titanium implant model.. J Biomed Mater Res A.

[13] Liang CT, Barnes J, Seedor JG, Quartuccio HA, Bolander M (1992). Impaired bone activity in aged rats: alterations at the cellular and molecular levels.. Bone.

[14] Suva LJ, Seedor JG, Endo N, Quartuccio HA, Thompson DD (1993). Pattern of gene expression following rat tibial marrow ablation.. J Bone Miner Res.

[15] Bab IA (1995). Postablation bone marrow regeneration: an in vivo model to study differential regulation of bone formation and resorption.. Bone.

[16] Zhang Q, Cuartas E, Mehta N, Gilligan J, Ke HZ (2008). Replacement of bone marrow by bone in rat femurs: the bone bioreactor.. Tissue Eng Part A.

[17] Sumner DR, Virdi AS, Leven RM, Healy KE, Shanbhag A, Rubash HE, Jacobs JJ (2006). Enhancing cementless fixation.. Joint replacements and bone resorption: pathology, biomaterials and clinical practice.

[18] Chung EH, Gilbert M, Virdi AS, Sena K, Sumner DR (2006). Biomimetic artificial ECMs stimulate bone regeneration.. J Biomed Mater Res.

[19] De Ranieri A, Virdi AS, Kuroda S, Shott S, Dai Y (2005). Local application of rhTGF- β2 modulates dynamic gene expression in a rat implant model.. Bone.

[20] Bais M, McLean J, Sebastiani P, Young M, Wigner N (2009). Transcriptional analysis of fracture healing and the induction of embryonic stem cell-related genes.. PLoS ONE.

[21] Simon R, Lam A, Li MC, Ngan M, Menenzes S (2007). Analysis of Gene Expression Data Using BRB-Array Tools.. Cancer Inform.

[22] Irizarry RA, Bolstad BM, Collin F, Cope LM, Hobbs B (2003). Summaries of Affymetrix GeneChip probe level data.. Nucleic Acids Res.

[23] Gautier L, Cope L, Bolstad BM, Irizarry RA (2004). affy–analysis of Affymetrix GeneChip data at the probe level.. Bioinformatics.

[24] Edgar R, Domrachev M, Lash AE (2002). Gene Expression Omnibus: NCBI gene expression and hybridization array data repository.. Nucleic Acids Res.

[25] Ramoni MF, Sebastiani P, Kohane IS (2002). Cluster analysis of gene expression dynamics.. Proc Natl Acad Sci U S A.

[26] Hosack DA, Dennis G, Sherman BT, Lane HC, Lempicki RA (2003). Identifying biological themes within lists of genes with EASE.. Genome Biol.

[27] Ashburner M, Ball CA, Blake JA, Botstein D, Butler H (2000). Gene ontology: tool for the unification of biology. The Gene Ontology Consortium.. Nat Genet.

[28] Kanehisa M, Goto S (2000). KEGG: kyoto encyclopedia of genes and genomes.. Nucleic Acids Res.

[29] Salomonis N, Hanspers K, Zambon AC, Vranizan K, Lawlor SC (2007). GenMAPP 2: new features and resources for pathway analysis.. BMC Bioinformatics.

[30] Eisen MB, Spellman PT, Brown PO, Botstein D (1998). Cluster analysis and display of genome-wide expression patterns.. Proc Natl Acad Sci U S A.

[31] Saldanha AJ (2004). Java Treeview–extensible visualization of microarray data.. Bioinformatics.

[32] Hamilton DW (2008). Functional role of periostin in development and wound repair: implications for connective tissue disease.. J Cell Commun Signal.

[33] Kashima TG, Nishiyama T, Shimazu K, Shimazaki M, Kii I (2009). Periostin, a novel marker of intramembranous ossification, is expressed in fibrous dysplasia and in c-Fos-overexpressing bone lesions.. Hum Pathol.

[34] Nah HD, Pacifici M, Gerstenfeld LC, Adams SL, Kirsch T (2000). Transient chondrogenic phase in the intramembranous pathway during normal skeletal development.. J Bone Miner Res.

[35] Itoh Y, Takamura A, Ito N, Maru Y, Sato H (2001). Homophilic complex formation of MT1-MMP facilitates proMMP-2 activation on the cell surface and promotes tumor cell invasion.. EMBO J.

[36] Zucker S, Drews M, Conner C, Foda HD, DeClerck YA (1998). Tissue inhibitor of metalloproteinase-2 (TIMP-2) binds to the catalytic domain of the cell surface receptor, membrane type 1-matrix metalloproteinase 1 (MT1-MMP).. J Biol Chem.

[37] Ries C, Egea V, Karow M, Kolb H, Jochum M (2007). MMP-2, MT1-MMP, and TIMP-2 are essential for the invasive capacity of human mesenchymal stem cells: differential regulation by inflammatory cytokines.. Blood.

[38] Sato H, Okada Y, Seiki M (1997). Membrane-type matrix metalloproteinases (MT-MMPs) in cell invasion.. Thromb Haemost.

[39] Stickens D, Behonick DJ, Ortega N, Heyer B, Hartenstein B (2004). Altered endochondral bone development in matrix metalloproteinase 13-deficient mice.. Development.

[40] Page-McCaw A, Ewald AJ, Werb Z (2007). Matrix metalloproteinases and the regulation of tissue remodelling.. Nat Rev Mol Cell Biol.

[41] Behonick DJ, Xing Z, Lieu S, Buckley JM, Lotz JC (2007). Role of matrix metalloproteinase 13 in both endochondral and intramembranous ossification during skeletal regeneration.. PLoS ONE.

[42] Kim JB, Leucht P, Lam K, Luppen C, Ten Berge D (2007). Bone regeneration is regulated by wnt signaling.. J Bone Miner Res.

[43] Zhong N, Gersch RP, Hadjiargyrou M (2006). Wnt signaling activation during bone regeneration and the role of Dishevelled in chondrocyte proliferation and differentiation.. Bone.

[44] Chen Y, Alman BA (2009). Wnt pathway, an essential role in bone regeneration.. J Cell Biochem.

[45] Takada I, Mihara M, Suzawa M, Ohtake F, Kobayashi S (2007). A histone lysine methyltransferase activated by non-canonical Wnt signalling suppresses PPAR-gamma transactivation.. Nat Cell Biol.

[46] Milat F, Ng KW (2009). Is Wnt signalling the final common pathway leading to bone formation?. Mol Cell Endocrinol.

[47] Satija NK, Gurudutta GU, Sharma S, Afrin F, Gupta P (2007). Mesenchymal stem cells: molecular targets for tissue engineering.. Stem Cells Dev.

[48] Etheridge SL, Spencer GJ, Heath DJ, Genever PG (2004). Expression profiling and functional analysis of wnt signaling mechanisms in mesenchymal stem cells.. Stem Cells.

[49] Baksh D, Boland GM, Tuan RS (2007). Cross-talk between Wnt signaling pathways in human mesenchymal stem cells leads to functional antagonism during osteogenic differentiation.. J Cell Biochem.

[50] Ling L, Nurcombe V, Cool SM (2009). Wnt signaling controls the fate of mesenchymal stem cells.. Gene.

[51] Baksh D, Tuan RS (2007). Canonical and non-canonical Wnts differentially affect the development potential of primary isolate of human bone marrow mesenchymal stem cells.. J Cell Physiol.

[52] Hilton MJ, Tu X, Wu X, Bai S, Zhao H (2008). Notch signaling maintains bone marrow mesenchymal progenitors by suppressing osteoblast differentiation.. Nat Med.

[53] Deregowski V, Gazzerro E, Priest L, Rydziel S, Canalis E (2006). Notch 1 overexpression inhibits osteoblastogenesis by suppressing Wnt/beta-catenin but not bone morphogenetic protein signaling.. J Biol Chem.

[54] Katoh M, Katoh M (2006). Notch ligand, JAG1, is evolutionarily conserved target of canonical WNT signaling pathway in progenitor cells.. Int J Mol Med.

[55] Katoh M, Katoh M (2006). NUMB is a break of WNT-Notch signaling cycle.. Int J Mol Med.

[56] Ying QL, Nichols J, Chambers I, Smith A (2003). BMP induction of Id proteins suppresses differentiation and sustains embryonic stem cell self-renewal in collaboration with STAT3.. Cell.

[57] Lind M, Overgaard S, Ongpipattanakul B, Nguyen T, Bünger C (1996). Transforming growth factor- β1 stimulates bone ongrowth to weight-loaded tricalcium phosphate coated implants.. J Bone Joint Surg.

[58] Sumner DR, Turner TM, Urban RM, Leven RM, Hawkins M (2001). Locally delivered rhTGF- β2 enhances bone ingrowth and bone regeneration at local and remote sites of skeletal injury.. J Orthop Res.

[59] Sena K, Sumner DR, Virdi AS (2010). Effect of recombinant human transforming growth factor-beta2 dose on bone formation in rat femur titanium implant model.. J Biomed Mater Res A J Biomed Mater Res A.

[60] Hankenson KD, Bornstein P (2002). The secreted protein thrombospondin 2 is an autocrine inhibitor of marrow stromal cell proliferation.. J Bone Miner Res.

[61] Delany AM, Hankenson KD (2009). Thrombospondin-2 and SPARC/osteonectin are critical regulators of bone remodeling.. J Cell Commun Signal..

[62] Pedemonte E, Benvenuto F, Casazza S, Mancardi G, Oksenberg JR (2007). The molecular signature of therapeutic mesenchymal stem cells exposes the architecture of the hematopoietic stem cell niche synapse.. BMC Genomics.

[63] Jeong JA, Ko KM, Bae S, Jeon CJ, Koh GY (2007). Genome-wide differential gene expression profiling of human bone marrow stromal cells.. Stem Cells.

[64] Wang WB, Fan JM, Zhang XL, Xu J, Yao W (2009). Serial expression analysis of liver regeneration-related genes in rat regenerating liver.. Mol Biotechnol.

[65] Greco JA, Pollins AC, Boone BE, Levy SE, Nanney LB (2009). A microarray analysis of temporal gene expression profiles in thermally injured human skin.. Burns.

[66] Velardo MJ, Burger C, Williams PR, Baker HV, Lopez MC (2004). Patterns of gene expression reveal a temporally orchestrated wound healing response in the injured spinal cord.. J Neurosci.

